# Physician attitudes towards—and adoption of—mobile health

**DOI:** 10.1177/2055207620907187

**Published:** 2020-02-20

**Authors:** Tracie Kong, Mary Morgan Scott, Yang Li, Cynthia Wichelman

**Affiliations:** 1 Fourth year medical students, Washington University School of Medicine, USA; 2 Department of Emergency Medicine, Washington University School of Medicine, USA

**Keywords:** Mobile health, digital medicine, smartphone apps, medical devices

## Abstract

**Objective:**

Smartphone apps and mobile devices are an emerging method of healthcare data collection. This study sought to understand how physicians currently view mobile health (mHealth) technologies and use them in patient care.

**Methods:**

A total of 186 physicians affiliated with Washington University School of Medicine in St. Louis, Missouri, USA completed a survey in 2016 regarding their current implementation of mHealth technologies for patient care and support for further development.

**Results:**

More than half of respondents were willing to discuss health apps and mobile devices with patients. However, most were not currently recommending them to patients. Apps/devices that encouraged a healthy diet and weight or tracked heart rate received the highest satisfaction ratings. Apps/devices that accessed the EMR (electronic medical record) remotely, provided medication reminders, or enrolled research subjects garnered the most interest despite respondents lacking prior experience. A majority agreed that collected biometrics are useful for promoting a healthy lifestyle (68%), tracking medical treatment (64%), or conducting research (56%); and agreed that proof of accuracy and precision (81%) and the efficient integration of collected data (68%) are necessary improvements. Uploading data automatically and updating physicians in real-time was the most preferred method of data integration into the EMR.

**Conclusions:**

Physicians show interest in using mHealth technologies for patient care but have limited experience, usually with those specific to their specialties. Proof of quality and a method to integrate data into the EMR are necessary for a mainstream role in healthcare.

## Introduction

Widespread use of mobile devices and increasing computing power has spurred the rise of mobile health (mHealth) technologies such as smartphone applications and activity trackers.^1,2^ An underdeveloped opportunity exists to integrate the unprecedented and accelerating stream of patient data collected from these technologies into medical practice.^3–6^ Devices have been developed to target various aspects of healthcare, such as encouraging healthy lifestyles, assisting with diagnoses, and improving patient care following treatment.^7–10^ When fully realized, mHealth has the potential to reduce costs, disseminate health information, extend care to resource-limited settings, and provide continuous information on individual biometrics to precisely diagnose and intervene in both acute and chronic disease.^11–15^

Yet, for physicians, the majority of these mobile apps and devices remains a novelty. There exists a paucity of guidelines for using the collected data for medical purposes.^16^ Studies on the effectiveness of apps are inconsistent and few in number.^17–19^ The FDA (Food and Drug Administration) has developed new policies adapted to the rapid development cycles of medical software to encourage technological progress while protecting patients.^20,21^ Under some of these regulatory frameworks, clearance is an assurance of safety rather than a marker of clinical utility.^22^ Apps with low risk for patient harm, such as activity trackers, are not within the FDA’s jurisdiction. Clinicians, as well as professional societies, are developing their own guidelines.^23,24^ Security and privacy of patient data remain an issue.^25,26^

In addition to these areas of concern, few studies have explored the perspective of physician adoption of mHealth technologies. In an interview series of 10 general practitioners in Australia, physicians reported benefits of mHealth apps including patient education and health recordkeeping, but the technology was not integrated into their workflow.^27^ In a survey of 50 general practitioners in Germany, physicians tended to support patients using mobile devices to keep track of medication use, weight, and blood pressure, while they disapproved of patients using mobile devices to look up medical information or assist with self-diagnosing.^28^ In a survey of 59 healthcare practitioners’ views on direct-to-consumer mobile teledermoscopy, some respondents noted the advantages of earlier skin cancer detection, but the majority were unsure or unconvinced that these devices should be provided to patients.^29^ A study on physicians’ perspectives towards mHealth in Turkey revealed that an innovation's perceived serviceability posed the greatest barrier to its implementation.^30^ Android and iOS app stores display ratings and number of downloads, but there is little data on how many physicians are recommending apps to their patients and which apps they recommend, if any. Overall, the perspective of physicians on mHealth technologies has not been well-researched.

To investigate physician attitudes towards the adoption of mHealth technologies, a survey of 1442 healthcare practitioners at Washington University School of Medicine was conducted to obtain insight into the best methods of approaching mHealth technologies and integrating them into medical practice. The survey queried physicians’ experience and satisfaction in using health apps and wearable devices, methods to integrate the data into the EMR, current implementation in their practices, and improvements that would support greater use of information collected by apps/wearables in their practices.

Survey results indicate that despite appreciable interest in mHealth technologies, few physicians recommended them to any of their patients at this time. Proof of accuracy and precision of the collected biometrics had the most potential to increase physician use of these technologies.

## Methods

The survey titled “Mobile apps and wearable/tracking devices in healthcare” was generated using Google Forms (Supplement). The survey was emailed to 1442 health professionals at Washington University School of Medicine, the majority of whom were physicians. The email contained a brief description of the study’s objectives and a link to the survey (Supplement). Physicians were asked to complete the survey within 23 days and received a reminder email one week before the deadline. The survey was estimated to take no longer than 5–10 minutes to complete. The participants completed the survey on their own accord and remained anonymous. No patient information or personal identifiers were disclosed, and no compensation was offered to participants.

The first of the three sections of the survey asked respondents to identify their area of practice, to describe their medical practice, to estimate the percentage of time dedicated to research, and to estimate the percentage of time dedicated to clinical practice. The answer options for each question on this page were arranged in alphabetic or numeric sequence.

The second section asked respondents for information regarding their experience using mobile apps and wearable/tracking devices in their medical practice. To prevent scoring bias favoring items that appear early or late in a sequence, the order of the health technologies listed was shuffled across forms such that each respondent received a randomized sequence of apps and devices to evaluate.

The final section asked respondents questions regarding the implementation of mobile apps and wearable/tracking devices. Response choices were shuffled except for those following alphabetic or numeric sequences.

All questions were required to be completed in the survey except for “Which HEALTH APPS and personal WEARABLES/DEVICES, if any, do you use in your practice that have NOT been mentioned?” and for the “Comments” field. All the required questions on each page of the survey had to be completed before moving on to the next page.

Survey responses were tabulated onto a Microsoft Excel document. Microsoft Excel was used for all data analysis. Only completed surveys were included in the analysis.

## Results

Of the 1442 potential respondents, 186 physicians completed the survey (12.9% response rate). Table 1 reports the response rate by medical specialty.

**Table 1.** Number of respondents recommending mobile health technologies to patients.

	Total number of respondents	Open to discussing apps with patients^a^	Recommend apps to patients
Anesthesiology	10	4 (40%)	3 (30%)
Cardiology	4	3 (75%)	0 (0%)
Cardiothoracic surgery	3	3 (100%)	0 (0%)
Critical care	1	0 (0%)	0 (0%)
Dermatology	4	1 (25%)	0 (0%)
Emergency medicine	15	7 (47%)	2 (13%)
Endocrinology	5	5 (100%)	3 (60%)
Gastroenterology	7	3 (43%)	2 (29%)
Genetics	2	1 (50%)	1 (50%)
General surgery	5	4 (80%)	1 (20%)
Geriatrics/nutrition	2	2 (100%)	1 (50%)
Hematology	1	0 (0%)	0 (0%)
Hospice and palliative medicine	1	0 (0%)	0 (0%)
Hospital medicine	13	4 (31%)	2 (15%)
Infectious disease	10	4 (40%)	0 (0%)
Internal medicine – general	1	0 (0%)	1 (100%)
Interventional radiology	2	2 (100%)	0 (0%)
Nephrology	2	1 (50%)	1 (50%)
Neurology	7	3 (43%)	0 (0%)
Neurosurgery	1	1 (100%)	0 (0%)
Obstetrics/gynecology	6	5 (83%)	2 (33%)
Oncology	8	3 (38%)	1 (13%)
Ophthalmology	4	1 (25%)	1 (25%)
Orthopedic surgery	8	3 (38%)	0 (0%)
Otolaryngology	3	3 (100%)	1 (33%)
Pain medicine	1	1 (100%)	1 (100%)
Pathology	1	0 (0%)	0 (0%)
Pediatrics	28	12 (43%)	4 (14%)
Physical medicine and rehabilitation	1	1 (100%)	0 (0%)
Plastic surgery	1	0 (0%)	0 (0%)
Psychiatry	13	9 (69%)	4 (31%)
Pulmonology	3	2 (67%)	2 (67%)
Radiology	7	4 (57%)	0 (0%)
Rheumatology	2	2 (100%)	0 (0%)
Sleep medicine	2	1 (50%)	1 (50%)
Urology	2	0 (0%)	0 (0%)
Total	186	95 (51%)	34 (18%)

a Respondents who indicated “agree” or “strongly agree” to the statement, “I am open to discussing the use of health apps and wearable/tracking devices with my patients.”*Note:* Percentages are of those who responded affirmatively out of the total respondents within each specialty.

### Openness to apps and devices

More than half of respondents agreed that they were open to discussing the use of health apps and wearable/tracking devices with patients (Table 1). Physicians recognized the potential of these technologies to maintain and re-establish physical activities. A rheumatologist stated that for patients with chronic pain syndrome, increased activity is the single most important predictor of treatment success. An emergency medicine physician wrote that of the plethora of disease processes seen in the ER, many are linked to obesity. A hepatologist stressed that patients need to maintain or increase activity prior to liver transplant as well as post-transplantation. Physicians in cardiology and geriatrics agreed that physical activity needs to be encouraged through goal-setting and self-motivation. An internist reported that they counsel patients on daily activity, and a neurologist wrote that the devices could be used to ensure return to activity after treatment.

Within the specialties of medical genetics, geriatrics/nutrition, otolaryngology, and pain medicine, at least one physician reported recommending apps and devices to 60% or more of their patients. In anesthesiology, endocrinology, obstetrics/gynecology, pulmonology, and psychiatry, a few physicians reported recommending health apps to at least 20% of their patients. However, the majority of physicians reported that they currently do not recommend these technologies to any of their patients.

### Reported satisfaction with apps and devices

**Figure 1.**
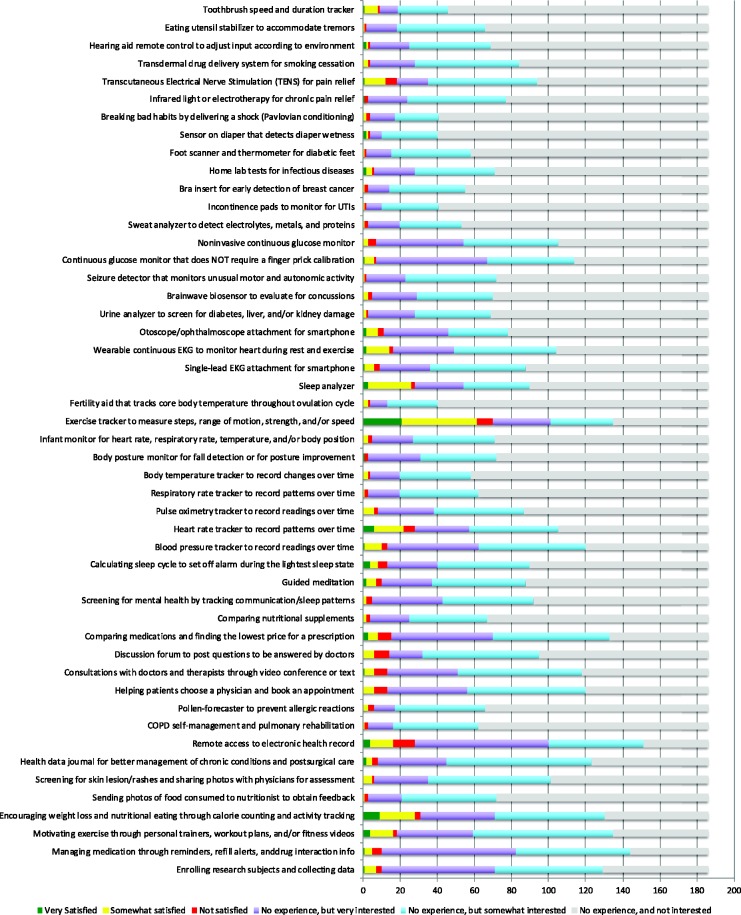
Ratings of mobile health technologies for patients by physicians. For each mHealth app, physicians rated their satisfaction level if they had prior experience with the app or their interest level in learning more about the app if no prior experience.

Physicians were asked to express their level of experience, satisfaction and interest towards a variety of mHealth smartphone applications or personal devices for patients (Figure 1). Respondents reported the most experience with “Exercise tracker to measure steps, range of motion, strength, and/or speed” (38%). The majority of those with experience using these exercise trackers in clinical care reported their experience as “Very satisfied” (30%) or “Somewhat satisfied” (57%).

**Table 2.** Ways mobile health technologies are recommended to patients.

	Respondents (%)
Have literature about app/device for patients	19 (10)
Prescribe the app/device to patients	18 (10)
Use app/device during patient visit	17 (9)
Have app/device in office to demonstrate to patients	15 (8)
Request for patients to purchase app/device and return with results	14 (7)
Have device to rent out to patients	3 (2)

For each of the other mHealth technologies listed in the survey, fewer than 20% of physicians reported prior experience. Respondents expressed highest satisfaction towards “Encouraging weight loss and nutritional eating through calorie counting, activity tracking, and/or personal emails” and “Heart rate tracker to record patterns over time.” Respondents expressed least satisfaction towards “Remote access to electronic health record” and “Discussion forum to post questions to be answered by doctors.”

At least a quarter of physicians responded they were very interested in the following apps despite lack of prior experience: “Remote access to electronic health record,” “Managing medication through dose reminders, medication diary, refill alerts, and/or drug interaction info,” “Enrolling research subjects and collecting data,” “Continuous glucose monitor that does NOT require a finger prick calibration,” “Comparing medications and finding the lowest price for a prescription,” and “Blood pressure tracker to record readings over time.”

Physicians also reported experience with medical apps and devices that were not included in the survey. These included Holter monitors and event monitors from an emergency medicine physician, radiograph viewing apps from a radiologist, seizure trackers from a neurologist, BiliTool from a pediatrician, and an app to track medical events in children with autism from a psychiatrist. In all, these responses indicate that apps with diverse functions designed to fit the clinical needs of specific medical specialties are available and useful in practice.

While the physicians who participated in the survey spanned a wide spectrum of medical specialties (Table 1), the low response numbers within each category did not allow for statistical analyses of differences across specialties. Of note, physicians did express experience and interest in mHealth technologies outside of their respective specialties. For example, a home lab test for infectious diseases garnered a high interest level in five infectious disease physicians but also in 17 physicians from other specialties, including nephrology, genetics, cardiothoracic surgery, anesthesiology, and radiology.

### Recommending apps and devices to patients

Physicians who already use health apps and devices in their practice reported mixed approaches for recommending and using these technologies for patient care (Table 2). At least 10% of respondents endorsed each of the following schemes: having literature about the app/device for patients, prescribing the app/device, and using the app/device during a visit.

### Areas of development for apps and devices

The survey also addressed potential improvements that would facilitate implementation of mHealth technologies (Table 3). A majority of respondents agreed that data from health apps and wearable/tracking devices are useful for promoting a healthy lifestyle (68%), tracking medical treatment (64%), or conducting research (56%). A significant minority also endorsed usefulness for making medical diagnoses (28%) or preventing disease (24%). A majority of respondents agreed that “proof of accuracy and precision” (81%) and “efficient integration of data collected” (68%) are valuable directions for improvement.

**Table 3.** Areas of development for apps and devices.

	Number of respondents
*What would enhance the likelihood that you would implement information collected by wearable/tracking devices in your practice? ^a^*	
Proof of accuracy and precision in biometrics collected	151 (81%)
Efficient integration of data collected into the EMR	127 (68%)
Involvement of physicians in developing and/or reviewing devices/apps	80 (43%)
Stringent regulation of how the data collected are stored, used, and shared	70 (38%)
Education to physicians on available devices/apps	69 (37%)
Technology for a single device to collect data on multiple aspects of a patient’s health	61 (33%)
FDA or other centralized regulation of devices/apps	60 (32%)
More patients using the devices/apps	53 (28%)
*Please rate the way(s) to collect information from patients' health apps and wearable/tracking devices from least effective (1) to most effective (5) ^b^*	
Patients use an application that automatically uploads data into the patient’s electronic medical record via a unique identifier, providing physicians with real-time updates.	82 (44%)
Patients and physicians use the same application, which physicians open to view patient data.	39 (21%)
Patients sync data from wearable devices to their smartphones and then show data to their physicians at appointments.	23 (12%)
Patients upload data to a secure online server. Physicians download data from server.	20 (11%)
Patients manually enter data into their charts at appointments.	2 (1%)
*How do you think data from health apps and wearable/tracking devices could be used in your practice? * ^a^	
Promote healthy lifestyle	127 (68%)
Track treatment	119 (64%)
Conduct research	105 (56%)
Make diagnoses	53 (28%)
Prevent disease	45 (24%)
*Who should pay for the apps/devices? ^a^*	
Private insurance	122 (66%)
Patient	122 (66%)
Centers for Medicare & Medicaid Services	86 (46%)
Employer	26 (14%)
Hospital	13 (7%)
Physician	2 (1%)

a Respondents were asked to choose all that applied.

b Number of respondents who rated the method as most effective.*Note:* Percentages are of those who selected a given choice out of the total 186 participants of the survey.

### Data management by EMRs

Challenges for entering the information collected by mHealth apps and devices into EMRs include transferring data in a timely manner to physicians, standardizing the format of data presentation, and ensuring patient privacy. The most popular method to achieve this integration was for applications to automatically upload data into EMRs via a unique identifier, providing physicians with real-time updates (Table 3). Respondents also favored a method whereby patients and physicians use the same application that could be opened by physicians to view patient data.

### Funding for apps and devices

Prices of health apps and devices range from free of charge to thousands of dollars. The majority of physicians indicated that private insurance (66%) and the patients themselves (66%) should pay for the apps and devices (Table 3). Approximately half believed that Centers for Medicare & Medicaid Services (46%) should be a payer. Most disagreed that patients’ employers and hospitals should be payers.

## Discussion

This survey sought to characterize how physicians regard and use health apps and wearables/devices for patients. Their perspectives can guide developers and manufacturers of apps and devices in creating products that complement current healthcare practices.

Despite high interest in mHealth technologies, few physicians were recommending apps/devices to patients in their practice at the time of the survey. This pattern of sparse use despite appreciable interest amongst physicians was evident across nearly all areas of medical practice. On the other hand, the majority of physicians were open to discussing these technologies with patients and expressed optimism for leveraging mHealth to promote healthy lifestyles, track treatment, and conduct research. The age of smartphones has already changed the way patients and physicians exchange health data. mHealth technologies can provide longitudinal health guidance, extending the counseling, assessment, and treatment monitoring for patients outside of medical facilities.

With the expansion of available health data comes many challenges.^31^ Proof of accuracy of the biometrics collected was the most cited improvement that physicians indicated would increase their use of mHealth technologies for their patients, according to the survey. This reflects the consensus that patient protection is of paramount importance. For instance, Roche’s Accu-Chek Connect diabetes management app was recalled by the FDA for miscalculating amounts of insulin.^32^ Standardization of biometrics from mHealth apps and provision of evidence-based guidelines to translate these data into diagnoses and interventions would enhance the use of these technologies for patient care.^17,33^ Public platforms such as AppScript, which allows NHS clinicians to prescribe apps from the NHS Apps Library, can be a reliable and up-to-date source of information.^34^

Furthermore, more information is not always useful. The onus is on physicians to review the data to make clinical decisions. In the worst case scenario, signal fatigue, unreliable data, or transmission delay may obscure important information and lead to patient harm.^11^ The Health Insurance Portability and Accountability Act (HIPAA) does not extend to patient data generated outside healthcare settings, and big data begets concerns about cybersecurity. The recently FDA-cleared Airstrip ONE is HIPAA compliant, but this is an exception to the rule. Studies have found that the majority of health-related apps on the iOS and Android marketplaces pose data privacy and security concerns, including information manipulation and leaks to third parties.^25,26^ Finally, another area of development supported by many physicians was a way to efficiently integrate data from the apps/devices into the EMR. Physicians prefer to view data in real-time, which could be achieved if apps automatically upload data into the EMR. Creators of mHealth technologies should note the importance of an efficient and secure method to transfer collected patient data to physicians.

Since the survey results were collected in 2016, the FDA has adopted new regulations for mHealth technologies.^35^ The amended definition of a medical device in the Food, Drug, and Cosmetic Act excludes certain medical software functions, including administrative support, lifestyle guides, electronic patient records, and data display/storage.^33^ Software providing clinical decision support or patient decision support would be evaluated on an individual basis for its potential to cause patient harm.^36^ Most FDA-cleared medical apps and wearable devices, including AliveCor, MobiUS, glucose monitoring systems, and recently the Airstrip ONE interoperability platform, utilize the 510(k) pathway, which only requires demonstration of equivalence to a predicate device on the market. In addition, the new de novo classification process since 2017 allows devices with no legally marketed predicate device to be designated as Class I or II rather than Class III, thereby simplifying their review process.^37^ Since then, Apple Watch’s EKG and heart rhythm detector have been granted de novo classification.^38^ The FDA has also launched a pre-certification program or streamlined premarket review, now in its pilot phase.^36^

This study is limited in that most of survey participants worked at a quaternary care center and major academic center in the USA, so the results may not be generalizable to other settings. Private practices and community hospitals were not sampled. Also, there is inherent bias in that physicians already interested in mHealth technology were more likely to respond. Another limitation is that while this study was aimed at physicians’ use of mHealth technologies in a professional setting, it is possible that some of the respondents described their experiences with personal use of the apps. Of note, many of the comments submitted did support that respondents were describing their experiences using apps and wearables for patient care rather than for private use.

Since the completion of this survey, mHealth technologies have grown exponentially, heightening the importance of the results. Appropriate developments could augment the use of mHealth apps and wearable devices to achieve higher quality patient care and a more efficient healthcare system. If these challenges are overcome, mHealth can transform healthcare through ubiquitous interventions for behavioral changes such as lifestyle practices or treatment adherence, telemedicine, screening, patient education and access to information, continuous monitoring of patient conditions, home testing, and medical access for the developing world. This potential calls for new paradigms to regulate mHealth technologies, standardized metrics for evaluation, ensured protection of patient information, and the bringing together of patients and caregivers, developers, and physicians to guide progress.

## Conclusions

In this online survey of physicians, the majority reported interest in mHealth apps and wearable devices for patients, but proof of accuracy and efficient integration of patient-generated data into medical records are needed before widespread use of these technologies is attained.

## Supplemental Material

DHJ907187 Supplemental material - Supplemental material for Physician attitudes towards—and adoption of—mobile health Click here for additional data file. Supplemental material, DHJ907187 Supplemental material for Physician attitudes towards—and adoption of—mobile health by Tracie Kong, Mary Morgan Scott, Yang Li and Cynthia Wichelman in Digital Health
