# Supplementary material for: Physician attitudes towards—and adoption of—mobile health
Source: Digit Health. 2020 Feb 20;6:2055207620907187. doi: 10.1177/2055207620907187 (PMC7036486; doi:10.1177/2055207620907187)
Supplement: DHJ907187 Supplemental material - Supplemental material for Physician attitudes towards—and adoption of—mobile health [file DHJ907187_Supplemental_material.pdf]

Survey: Mobile apps and wearable/tracking devices in healthcare

Thank you for taking this survey on the applications of mobile apps and wearable/tracking devices in medical care and research.

1. Which best describes your area of practice? [Choose one]

- Allergy/immunology
- Anesthesiology
- Bone/mineral health
- Cardiology
- Cardiothoracic surgery
- Dermatology
- Emergency medicine
- Endocrinology
- Family medicine
- Gastroenterology
- General surgery
- Genetics
- Geriatrics/nutrition
- Hematology
- Hospital medicine
- Infectious disease
- Internal medicine - general
- Neurology
- Neurosurgery
- Obstetrics/gynecology
- Occupational therapy
- Oncology
- Ophthalmology
- Orthopedic surgery
- Otolaryngology
- Pathology
- Pediatrics
- Physical medicine and rehabilitation
- Physical therapy
- Plastic surgery
- Primary care
- Psychiatry
- Pulmonology
- Radiation oncology
- Radiology
- Renal
- Rheumatology
- Sleep Medicine
- Urology
- Other:

2. About what percent of your time is dedicated to research? [Choose one]

- 0%
- 20%
- 40%
- 60%
- 80%
- 100%

3. Which best describes your medical practice? [Choose one]
- Hospital-based practice
- Group practice
- Solo practice
- Military medicine
- Concierge medicine
- Other:

4. About what percent of your time is dedicated to clinical practice? [Choose one]
- 0%
- 20%
- 40%
- 60%
- 80%
- 100%

Functions of mobile apps and wearable/tracking devices

The following questions address different mobile HEALTH APPS and WEARABLE/TRACKING DEVICES that are available, either to aid physicians in their practice and research, or to track patient health and well-being. With each application or device, we describe the general uses followed by example(s) of such an app or device. There may be other examples not listed; please respond based on any app or device that is used in a similar fashion even if the example(s) listed may not be familiar to you.

5. Do you have experience working with HEALTH APPS in the following areas available for patients? If YES, please indicate your level of satisfaction with the app. If NO, please indicate your level of interest in having your patients use the app.

- Very Satisfied
- Somewhat satisfied
- Not satisfied
- No experience, but very interested
- No experience, but somewhat interested
- No experience, and not interested

[Order of apps was shuffled for each respondent.]

- Sending photos of food consumed to nutritionist to obtain feedback (e.g. Rise)
- Comparing nutritional supplements (e.g. LabDoor)
- Pollen-forecaster to prevent allergic reactions (e.g. Clarityn)
- Helping patients choose a physician and book an appointment (e.g. ZocDoc, HealthYI, Amino)

- Consultations with doctors and therapists through video conference or text (e.g. MDLive, Talkspace, HealthTap)
- Health data journal for better management of chronic conditions, postsurgical care, and/or mental health (e.g. Symple, SeamlessMD, Iodine's Start, HemMobile)
- Discussion forum to post questions to be answered by doctors (e.g. HealthTap)
- Remote access to electronic health record (e.g. iBlueButton, Capzule PHR)
- Screening for mental health by tracking communication patterns, sleep patterns, and/or visits to different places (e.g. Ginger.io, Purple Robot)
- Motivating exercise through personal trainers, workout plans, and/or fitness videos (e.g. Couch to 5K, Cody, Pact)
- Comparing medications and finding the lowest price for a prescription (e.g. Blink Health)
- Calculating sleep cycle to set off alarm during the lightest sleep state (e.g. Sleep Cycle)
- Managing medication through dose reminders, medication diary, refill alerts, and/or drug interaction info (e.g. Mango Health)
- COPD self-management and pulmonary rehabilitation based on medication history, daily symptoms, and other health data (e.g. MyCOPD)
- Screening for skin lesion/rashes and sharing photos with physicians for assessment (e.g. SkinVision)
- Enrolling research subjects and collecting data (e.g. Apple's ResearchKit)
- Guided meditation (e.g. Meditation Studio, Deep Sleep with Andrew Johnson)
- Encouraging weight loss and nutritional eating through calorie counting, activity tracking, and/or personal emails (e.g. My Diet Coach, MyFitnessPal, Kurbo Health)

6. Do you have experience working with the following personal TRACKING/WEARABLE DEVICES that collect biometric data? If YES, please indicate your level of satisfaction with the device in your practice. If NO, please indicate your level of interest in having your patients use the device.

- Very Satisfied
- Somewhat satisfied
- Not satisfied
- No experience, but very interested
- No experience, but somewhat interested
- No experience, and not interested

[Order of devices was shuffled for each respondent.]

- Fertility aid that tracks core body temperature throughout ovulation cycle (OvulaRing)
- Exercise tracker to measure steps, range of motion, strength, and/or speed (e.g. Fitbit, GymWatch, DigitSole shoes)
- Seizure detector that monitors unusual motor and autonomic activity (e.g. Embrace)
- Bra insert for early detection of breast cancer (e.g. Cyrcadia Health's iTBra)
- Continuous glucose monitor that does NOT require a finger prick calibration (e.g. Abbott's FreeStyle Libre)
- Infant monitor for heart rate, respiratory rate, temperature, and/or body position (e.g. Owlet's smart sock, Mimo, Sproutling, Exmobaby)
- Body temperature tracker to record changes over time (e.g. HealthPatch MD)
- Wearable continuous EKG to monitor heart during rest and exercise (e.g. QardioCore, CardioScape Holter Monitor)
- Hearing aid remote control to adjust input according to environment (e.g. ReSound, Sivantos)

- Otoscope/ophthalmoscope attachment for smartphone to enable photo/video recordings of ear infections (e.g. Seymour, CellScope)
- Sleep analyzer (e.g. Beddit, Sleep Image, Fitbit)
- Infrared light or electrotherapy for chronic pain relief (e.g. LumiWave)
- Heart rate tracker to record patterns over time (e.g. Apple Watch, Sona Connected Bracelet)
- Respiratory rate tracker to record patterns over time (e.g. Spire, HealthPatch MD)
- Urine analyzer to screen for diabetes, liver, and/or kidney damage (e.g. uChek)
- Sweat analyzer to detect electrolytes, metals, and proteins (e.g. Kenzen ECHO Smart Patch, continuous sweat measuring bracelet developed at UC Berkeley)
- Pulse oximetry tracker to record readings over time (e.g. WristOx2, MocaHeart)
- Eating utensil stabilizer to accommodate tremors (e.g. Liftware)
- Noninvasive continuous glucose monitor (e.g. Google's contact lens, Sano)
- Foot scanner and thermometer for diabetic feet (e.g. Podimetrics)
- Toothbrush speed and duration tracker (e.g. Beam Dental)
- Breaking bad habits by delivering a shock (Pavlovian conditioning) (e.g. Pavlok)
- Brainwave biosensor to evaluate for concussions (e.g. Cerora's headset)
- Body posture monitor for fall detection or for posture improvement (e.g. HealthPatch MD, Lumo Lift)
- Sensor on diaper that detects diaper wetness (e.g. Huggies TweetPee)
- Home lab tests for infectious diseases (e.g. 15-minute HIV and syphilis test developed at Columbia University)
- Transcutaneous Electrical Nerve Stimulation (TENS) for pain relief (e.g. Quell, HealthmateForever)
- Transdermal drug delivery system for smoking cessation (e.g. Chrono Therapeutics)
- Blood pressure tracker to record readings over time (e.g. Omron, Withings Wireless BP Monitor)
- Incontinence pads to monitor for UTIs (e.g. Pixie Scientific's Pixie Pads)
- Single-lead EKG attachment for smartphone (e.g. AliveCor Kardia)

7. Which HEALTH APPS and personal WEARABLES/DEVICES, if any, do you use in your practice that have NOT been mentioned? Please indicate your satisfaction with each app: satisfied, somewhat satisfied, or dissatisfied

[Free response]

Implementation of mobile apps and wearable/tracking devices

8. How do you think data from health apps and wearable/tracking devices could be used in your practice (choose all that apply)? [Order of choices was shuffled for each respondent.]
- Conduct research
- Promote healthy lifestyle
- Prevent disease
- Make diagnoses
- Track treatment
- Other:

9. I am open to discussing the use of health apps and wearable/tracking devices with my patients.
(1=strongly disagree to 5=strongly agree)

10. What would enhance the likelihood that you would implement information collected by wearable/tracking devices in your practice (choose all that apply)? [Order of choices was shuffled for each respondent.]
- Efficient integration of data collected into the electronic medical record

- Stringent regulation of how the data collected is stored, used and shared
- FDA or other centralized regulation of devices/apps
- More patients using the devices/apps
- Proof of accuracy and precision in biometrics collected
- Involvement of physicians in developing and/or reviewing devices/apps
- Technology for a single device to collect data on multiple aspects of a patient's health
- Offer education to physicians on available devices/apps, such as through videos or in person
- Other:

11. Please rate the way(s) to collect information from patients' health apps and wearable/tracking devices from least effective (=1) to most effective (=5). [Order of choices was shuffled for each respondent.]

- Patients manually enter data into their charts at appointments.
- Patients sync data from wearable devices to their smartphones and then show data to their physicians at appointments.
- Patients and physicians use the same application, which physicians open to view patient data.
- Patients upload data to a secure online server. Physicians download data from server.
- Patients use an application that automatically uploads data into the patient's electronic medical record via a unique identifier, providing physicians with real-time updates.

12. If you are using apps/wearable devices in your practice, how are you using them (choose all that apply)? [Order of choices was shuffled for each respondent.]

- Request for patients to purchase and return with results
- Have app/device in office to demonstrate to patients
- Have device to rent out to patients
- Have literature on app/device for patients
- Prescribe the app/device for patients
- Use app/device during patient visit
- Other:

13. What percent of your patients do you recommend apps for? [Choose one]

- 0%
- 20%
- 40%
- 60%
- 80%
- 100%

14. What are the top 3 apps you recommend? [Free response]

15. What percent of your patients do you recommend wearable devices for? [Choose one]

- 0%
- 20%
- 40%
- 60%
- 80%
- 100%

16. What are the top 3 wearable devices you recommend? [Free response]

17. Who should pay for the apps/devices (choose all that apply)?

- Private insurance
- Centers for Medicare & Medicaid Services
- Patient
- Physician
- Hospital
- Employer
- Other:

18. Comments: [Free response]
